# Complete Genome Sequence of *Cedecea neteri* Strain SSMD04, a Bacterium Isolated from Pickled Mackerel Sashimi

**DOI:** 10.1128/genomeA.01339-14

**Published:** 2014-12-18

**Authors:** Kok-Gan Chan, Kian-Hin Tan, Wai-Fong Yin, Jia-Yi Tan

**Affiliations:** Division of Genetics and Molecular Biology, Institute of Biological Sciences, University of Malaya, Kuala Lumpur, Malaysia

## Abstract

We report here the complete genome sequence of *C. neteri* SSMD04, a strain isolated from pickled mackerel sashimi, sequenced by third-generation sequencing technology. To the best of our knowledge, this is the first documentation that reports the complete genome of *Cedecea neteri*.

## GENOME ANNOUNCEMENT

*Cedecea* is a genus of Gram-negative rod-shape bacteria that belongs to the *Enterobacteriaceae* family (1). This genus of bacteria is characterized by lipase positivity and resistance to colistin and cephalothin (2). Isolation of *Cedecea* is rare, and its discovery was originally from a human source (2). Even though the members of *Cedecea* have been suspected to be human pathogens, their pathogenicity is largely unknown. This is because most of the *Cedecea* spp. have been isolated from polymicrobial and severely immunocompromised conditions, making the assessment of their medical importance difficult (3). The first evidence of *Cedecea* spp. as human pathogens was discovered in the cases of *C. neteri* infection, in which the patients were diagnosed with possible endocarditis and systemic lupus erythematosus. Complications from bacteremia of the second case even led to the patient’s death (4, 5).

*Cedecea* strains were mostly isolated from a human source but not exclusively. There were cases in which this bacterium has been isolated from fruit flies (6) and vegetables (7). Here, we report the complete genome of *C. neteri* strain SSMD04, isolated from pickled mackerel (*Shime saba*) sashimi.

The whole genome of *C. neteri* SSMD04 was sequenced with a Pacific Biosciences single-molecule real-time (PacBio SMRT) sequencer (8, 9). A 20-kb SMRTbell library was prepared, and the sequencing was carried out using P5 chemistry on two SMRT cells. *De novo* assembly of 24,369 reads using the hierarchical genome assembly process (HGAP) algorithm in the SMRT version 2.1.1 portal (10) resulted in a single contig of 4.88 Mb in size. The genome has a GC content of 55.1% with an average coverage of 38.42×. This genome was then annotated by RAST (Rapid Annotation using Subsystem Technology) version 2.0 (11), which successfully identified 4,472 coding sequences, as well as 103 RNA sequences. Of these, 56.64% of the annotated coding sequences fell within 539 subsystems available in the RAST database. Similar to other bacteria, most of the genes are involved in common cell metabolism that sustains the survival of *C. neteri* SSMD04 itself, where 583 gene counts were attributed to carbohydrates, 502 to amino acids and derivatives, 325 to protein metabolism, as well as 247 to cofactors, vitamins, prosthetic groups, and pigments. We hope that the complete genome sequence of this isolate will facilitate the discovery of various genes and their functions in this isolate.

### Nucleotide sequence accession numbers.

This complete genome project has been deposited at DDBJ/EMBL/GenBank under the accession number CP009451. The version described in this paper is the first version, CP009451.1.
